# Climate change and the growing threat of bunyaviruses

**DOI:** 10.1128/jvi.01772-25

**Published:** 2026-07-16

**Authors:** James M. Bowen, Natasha L. Tilston

**Affiliations:** 1 Department of Microbiology and Immunology, Indiana University School of Medicine 734638, Indianapolis, Indiana, USA; Indiana University Bloomington, Bloomington, Indiana, USA

**Keywords:** climate change, bunyaviruses, vector competence, viral reassortment, arthropod vectors, emerging and reemerging viruses

## Abstract

Anthropogenic climate change and its implications for infectious diseases have been central to the scientific and public health discourse for more than three decades. In 2026, we have now entered a climate adaptation phase, with accumulating evidence demonstrating that ongoing climatic variability is directly reshaping the dynamics of multiple vector-borne viruses, including bunyaviruses. Bunyaviruses comprise a diverse group of segmented, negative-sense RNA viruses that include significant human and veterinary pathogens, such as Rift Valley fever virus, Crimean-Congo hemorrhagic fever virus, Oropouche virus, and Cache Valley fever virus. Despite their medical and veterinary importance, climate-virus relationships remain comparatively less well characterized for bunyaviruses than for viruses such as dengue, Zika, chikungunya, and yellow fever. This gap is increasingly notable in light of recent epidemiological shifts, exemplified by the 2022–2025 expansion of Oropouche virus across the Americas and the Caribbean, which highlights how bunyaviruses can exploit ecological disruption and underscores the need to better understand the environmental determinants of their transmission. Advances in genomic surveillance, experimental virology, and climate-informed epidemiology now enable these interconnected virus-host-vector-environment dynamics to be examined with increasing precision. In this minireview, we synthesize current evidence linking environmental change to bunyavirus transmission and geographic expansion, identify critical mechanistic and surveillance gaps, and outline priorities for developing predictive frameworks to strengthen bunyavirus preparedness within a One Health context.

## INTRODUCTION

Seventeen years have passed since Richard M. Elliott published his review of bunyaviruses and climate change (1). In that article, Richard offered a prescient and measured assessment of how environmental change might influence bunyavirus emergence. He cautioned against attributing outbreaks solely to climate and instead emphasized the complex interplay among viral evolution, vector competence, vector ecology, and environmental factors. That central message remains fundamentally true. What has changed, however, is the global climate context. Anthropogenic warming is now unequivocal, and its biological consequences are increasingly measurable. When Richard’s paper was published in 2009, climate-sensitive infectious diseases were largely framed within an awareness-and-early-planning paradigm (2). Observed signals of climate-driven change were limited, long-term data sets were sparse, and climate was viewed as one driver among many, alongside poverty, urbanization, land-use change, and health system capacity (3). Today, that framing has shifted. The Sixth Assessment Report of the Intergovernmental Panel on Climate Change (IPCC) treats infectious diseases as a major and already occurring health impact of climate change, supported by stronger causal attribution, expanded geographic evidence, and projections of increasing future burden (4, 5). This broader reframing is reinforced by quantitative analyses demonstrating the scale of climate-pathogen interactions (6). Mora et al. estimated that 58% of known human infectious diseases have been aggravated by climatic hazards, with more than 200 pathogens affected by at least one climate-related factor. Among these, vector-borne diseases emerge as particularly sensitive to environmental change, as temperature shifts, altered precipitation, and ecosystem disruption modify the distribution, abundance, and seasonal activity of mosquitoes, ticks, midges, birds, and rodent hosts. In this evolving landscape, bunyaviruses, many of which depend on environmentally responsive vectors, are positioned to exploit ecological disruption in ways that were not fully appreciable in 2009.

This review revisits the question posed nearly two decades ago: how does climate change shape the emergence of bunyaviruses? While Richard’s review defined key molecular and biological determinants of bunyavirus transmission, here we shift focus to the ecological and environmental contexts that govern when, where, and how these viruses emerge. We examine how environmental change reshapes transmission dynamics, geographic expansion, and epidemic potential, and consider how integrating ecological understanding with surveillance can inform anticipatory public health strategies.

## GENOME ARCHITECTURE AND EVOLUTIONARY PLASTICITY OF BUNYAVIRUSES

Bunyaviruses have undergone substantial taxonomic reorganization over the past decade. Beginning in the mid-2010s, the family *Bunyaviridae* was elevated to the order *Bunyavirales* (7, 8) and significantly expanded to accommodate newly characterized viruses. In April 2024, *Bunyavirales* was further elevated to the class *Bunyaviricetes* (9), which now comprises the orders *Elliovirales* (named in honor of Richard), which encompasses the “classic” arthropod- and rodent-borne bunyaviruses: orthobunyaviruses, hantaviruses, and tospoviruses, and *Hareavirales*, which includes arenaviruses and the remaining bunyaviruses: nairoviruses and phenuiviruses (7, 8, 10–12). As a result of this reclassification, the historical distinction between “classic” bunyaviruses and arenaviruses has become taxonomically blurred, and these terms are now used largely in a colloquial sense to refer to viruses with three- or two-segmented genomes, respectively. For clarity and continuity with much of the prior literature, this review focuses on the three-segmented members historically recognized as bunyaviruses. While most medically relevant bunyaviruses possess tripartite genomes, it is important to note that greater genomic diversity exists within the class, particularly among plant-infecting bunyaviruses, including members of the *Coguvirus* (two segments) and *Tenuivirus* (four segments) genera (family *Phenuiviridae*), and the *Emaravirus* genus (six segments; family *Fimoviridae*) (13). These viruses are not considered further here.

Bunyaviruses are enveloped, spherical virions approximately 100 nm in diameter that possess a tripartite, negative- or ambisense RNA genome comprising the small (S), medium (M), and large (L) segments. The S segment encodes the nucleoprotein (N) and, in many genera, a nonstructural protein (NSs). The M segment encodes a glycoprotein precursor that is post-translationally processed into the envelope glycoproteins Gn and Gc and, in some viruses, an additional nonstructural protein (NSm). The L segment encodes the viral RNA-dependent RNA polymerase. Each coding region is flanked by untranslated regions that contain signals essential for transcription, replication, and genome packaging (12, 14–16).

**Fig 1:**
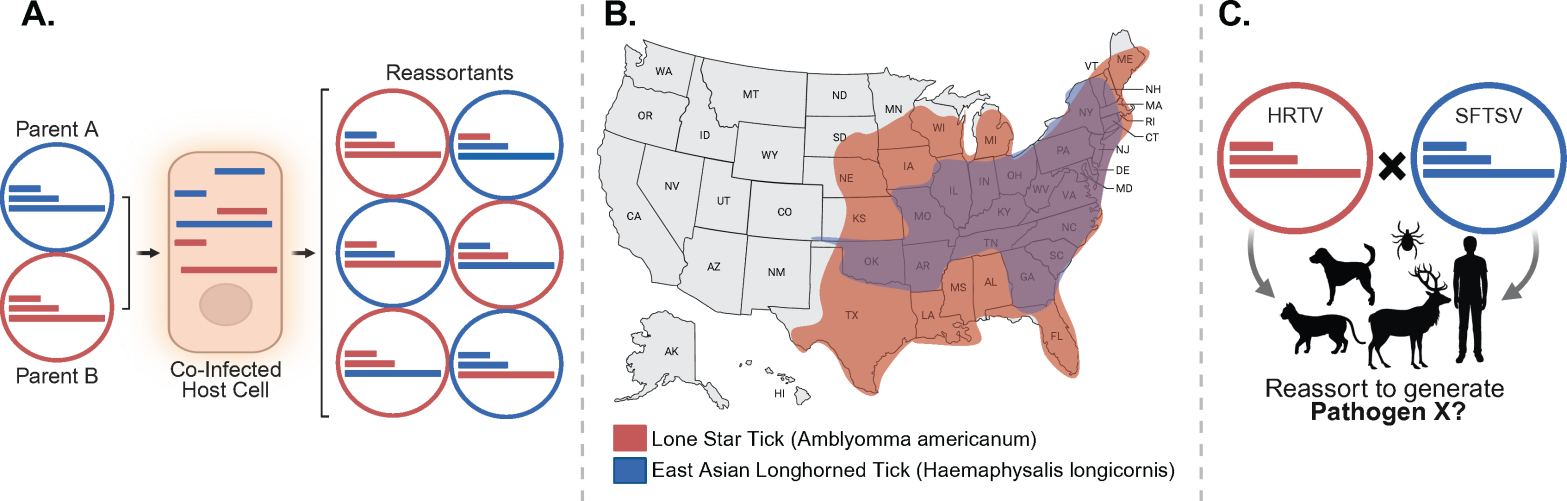
Hypothetical reassortment between HRTV and SFTSV. (**A**) Schematic representation of six possible reassortant progeny upon co-infection of tri-segmented bunyaviruses. (**B**) Overlayed distribution of tick species *A. americanum* (23) and *H. longicornis* (24) within the United States. (**C**) Reassortment between HRTV and SFTSV could generate emergent pathogens. Created in BioRender. Tilston-Lunel, N. (2026) https://BioRender.com/yx5dk9q.

A defining biological feature of bunyaviruses is their segmented genome, which permits genetic reassortment during co-infection and the rapid generation of novel genotypic combinations with altered phenotypic properties (12, 14, 17–19). Since reassortment depends on the co-circulation of related viruses within shared vector and host populations, ecological shifts that alter vector distribution, density, and species overlap may increase opportunities for segment exchange (Box 1; Fig. 1). Most bunyaviruses are transmitted by arthropod vectors, including mosquitoes, ticks, thrips, midges, and sandflies, and are maintained in transmission cycles that are highly sensitive to environmental conditions (4, 5, 12, 14, 20–22). Hantaviruses are a notable exception, as they are transmitted primarily through exposure to infected rodent excreta and are maintained in rodent reservoir populations (12, 14, 22). Consequently, climatic changes that reshape vector habitats and transmission networks have the potential not only to expand the geographic range of bunyaviruses but also to influence their evolutionary dynamics (19).

Box 1. **A plausible reassortment scenario in emerging bunyaviruses**

Segmented RNA viruses can exchange entire genome segments during co-infection, generating new genetic constellations with potentially altered phenotypes (Fig. 1A). The likelihood of such events depends not only on viral compatibility but also on ecological conditions that bring related viruses into shared transmission cycles.

A scenario of increasing relevance involves the convergence of two tick-borne bandaviruses: Heartland virus (HRTV), found in the United States (U.S.), and severe fever with thrombocytopenia syndrome virus (SFTSV), which circulates widely in East Asia. These viruses are phylogenetically related and cause a similar thrombocytopenic disease but differ in severity.

Their vectors are distinct but increasingly overlapping. The lone star tick (*Amblyomma americanum*), which transmits HRTV, is broadly established across the eastern and central U.S. (23), while the Asian longhorned tick (*Haemaphysalis longicornis*), a known vector of SFTSV, has recently been detected in the U.S. and continues to expand its range (24). The increasing geographic overlap of these tick species, together with shared vertebrate hosts, creates plausible opportunities for co-infection (Fig. 1B). Moreover, climate-sensitive changes in tick activity, abundance, survival, seasonality, and habitat suitability may further expand opportunities for tick-host interactions to occur (4, 5, 20, 21). These changes may also affect wild hosts, such as white-tailed deer (*Odocoileus virginianus*), an important U.S. host for both tick species with an expanding northern range (25). Shifts in host abundance, tick distribution, and human contact are occurring in difficult-to-predict ways that could facilitate SFTSV establishment in the U.S.

Although reassortment between HRTV and SFTSV has not been documented, their genetic relatedness and converging ecological niches raise the possibility that reassortants could arise if these viruses co-circulate, as supported by *in vitro* experiments (17, 18). Such an event could alter transmission dynamics, host range, or virulence, underscoring how environmental change can generate opportunities for the emergence of new pathogens (Fig. 1C).

## CLIMATE-SENSITIVE ECOLOGICAL INTERFACES IN BUNYAVIRUS EMERGENCE

Bunyavirus history has been marked by recurrent outbreaks, rapid geographic spread, and the emergence of new viral lineages, sometimes through reassortment. These dynamics occur within shifting ecological contexts shaped by interactions among host, vector, and environmental systems, each of which responds to climate change in distinct but interconnected ways.

### Host-dependent transmission interfaces

#### Rodents (direct reservoir interface)

The first and arguably most impactful animal hosts associated with climate-sensitive bunyavirus transmission are rodents. Members of the genus *Orthohantavirus* are etiological agents of two significant human diseases: hantavirus pulmonary/cardiopulmonary syndrome (HPS/HCPS), associated with New World hantaviruses, and hemorrhagic fever with renal syndrome, caused by Old World hantaviruses. Unlike arthropod-borne bunyaviruses, hantaviruses are transmitted primarily through inhalation of aerosolized rodent excreta, and there are no known arthropod vectors. Human-to-human transmission is limited to strains of Andes virus (ANDV), which cause high-mortality HCPS across Chile and Argentina (26–29). Though this became a major topic of concern during the 2026 ANDV outbreak aboard the *MV Hondius* cruise ship (30–32), human-to-human transmission of ANDV appears to be exceedingly rare, requiring close, prolonged, or intimate contact with a symptomatic individual (29, 33). Consequently, viral maintenance and dispersal are directly dependent on rodent population dynamics (34–36). Climate-driven shifts in rodent abundance, diversity, and behavior therefore have immediate consequences for hantavirus transmission in endemic regions (34, 36–39). A prominent example is the emergence of New World hantaviruses in the southwestern United States (U.S.). Although Old World hantaviruses, such as Seoul virus, had been identified previously (40, 41), the identification of Sin Nombre virus (SNV) in 1993 marked the first of numerous New World hantaviruses discovered across North, Central, and South America in the following decades (35, 42–44). Although retrospective cases have been suspected as early as 1959 (45–47), SNV was first reported in the U.S. only during a large-scale outbreak in the arid Four Corners region. This outbreak occurred after unusually heavy precipitation associated with the 1992–1993 El Niño event, which triggered a surge in vegetation growth and food availability, leading to a rapid expansion of rodent populations and increased human exposure (37, 48). Similar precipitation-driven amplification events have been observed in subsequent outbreaks (49). However, the relationship between climate, rodent population, and virus transmission is complex and not strictly linear. Reductions in biodiversity have been linked to increased hantavirus transmission (36). In parallel, habitat disruption (e.g., drought, wildfire) can alter rodent community composition and drive rodent migration into peri-urban or urban settings, intensifying the rodent-human interface and increasing transmission risk. Together, these patterns illustrate how climate-sensitive changes in reservoir ecology can drive bunyavirus emergence through both amplification and displacement (36, 50–53).

#### Avian hosts (dispersal interface)

In contrast to rodent-driven amplification systems, migratory birds contribute to bunyavirus emergence primarily through long-distance vector dispersal. Crimean-Congo hemorrhagic fever virus (CCHFV) has repeatedly been detected in *Hyalomma* ticks removed from migratory passerines along the Afro-Eurasian flyways (54, 55), implicating birds in transcontinental virus movement. Surveillance studies at Mediterranean migratory stopover sites in Morocco, Greece, and Italy have identified CCHFV RNA in immature *Hyalomma marginatum* complex ticks collected from passerines, with phylogenetic analyses demonstrating clustering with African genotypes, consistent with trans-Saharan introduction (54, 55). Immature *Hyalomma marginatum* and *Hyalomma rufipes* preferentially parasitize birds, particularly passerines, allowing larvae acquired in sub-Saharan Africa to remain attached during migration and detach as infected nymphs in southern Europe. Since most avian species do not develop detectable viremia sufficient to infect feeding ticks, these birds function primarily as mechanical disseminators of infected ticks rather than amplifying hosts (55, 56). This is especially relevant for *H. marginatum*, as they often prefer to remain attached to a single host during the feeding period of both the larval and nymphal developmental stages (relatively uncommon among tick species), allowing ample opportunity (typically 12–26 days on the first host) for long-range transport by migratory birds before detaching as adults (57). Phylogeographic analyses support repeated African introductions into Europe, consistent with episodic CCHFV importation via avian-mediated tick transport (58). Ecological niche modeling further predicts that warming Mediterranean climates increasingly favor the establishment of competent *Hyalomma* vectors in southern and central Europe, suggesting a two-step emergence process: long-distance introduction via migratory birds, followed by local amplification in climatically permissive regions (57).

A similar dispersal pattern has been proposed for severe fever with thrombocytopenia syndrome virus (SFTSV), first described in China in 2009 (59–61). Since its emergence, SFTSV has expanded dramatically across mainland China (62, 63) and was subsequently reported in Taiwan in 2020 (64, 65), with incidence closely aligned with ecological suitability for ticks and hosts (63). Migratory birds are increasingly considered potential vehicles for long-range dispersal of infected *Haemaphysalis* ticks (65–67), reinforcing the role of avian movement in regional spread. Together, these findings support a climate-sensitive emergence model in which avian-mediated tick dispersal facilitates repeated long-distance introductions, while environmental suitability determines whether transient introductions progress to sustained transmission cycles.

#### Livestock and wild mammals (amplifying host interface)

Livestock and wild mammals connect vector, animal, and human transmission cycles. For mosquito-transmitted Rift Valley fever virus (RVFV), sheep, goats, and cattle typically develop high levels of viremia, enabling rapid within-herd spread that manifests as abortion storms and high mortality (68). These viremic herds increase mosquito infection, secondary transmission, and human spillover potential. Climate plays a key role in shaping these dynamics by influencing vector abundance, feeding behavior, and the distribution of primary and secondary mosquito species, thereby enhancing transmission within livestock populations. However, while climate determines ecological suitability, anthropogenic factors govern viral movement. Livestock are frequently transported through legal and illegal trade networks, as well as through pastoral movement between endemic and non-endemic regions (69). The emergence of RVFV in Egypt, with major outbreaks in 1977–1978, 1993, and 1997, illustrates this process, where repeated importation of infected animals, particularly from Sudan, into permissive ecological settings enabled local vector-driven amplification (70, 71). In addition to vector-borne transmission, direct exposure through consumption of raw or unpasteurized milk (72) and handling of raw meat (73–76) can contribute to endemic RVFV circulation. Together, these routes highlight that RVFV emergence is rarely a single spillover event but instead reflects sustained reintroduction through interconnected livestock systems, where increasing host density, diversity, and regional connectivity create continuous pathways for viral dissemination (77, 78). Similar processes underpin the emergence of CCHFV and Nairobi sheep disease virus (79), where changes in livestock management alter human exposure risk. For example, drought-driven shifts in grazing practices in West Africa brought livestock closer to urban centers, which in turn contributed to CCHFV emergence in the area (80).

These same climate and anthropogenic pressures extend beyond livestock to wild mammal populations, where changes in host abundance, distribution, and physiology reshape transmission dynamics. In North America, climate change has driven the expansion of white-tailed deer populations through milder winters and increased survival and reproductive success (25). This expansion, reinforced by land-use change and reduced hunting pressure, has increased deer densities and extended their range into peri-urban and previously unsuitable regions, enhancing contact between hosts and vectors (60). Experimental studies among orthobunyaviruses have shown that white-tailed deer are competent amplifying hosts of Potasi virus, Jamestown Canyon virus, and Cache Valley virus (CVV), capable of infecting mosquitoes and sustaining transmission cycles (81). Consistent with this, a high seroprevalence of these viruses has been found across the states of Indiana and North Carolina, indicating widespread exposure among the wild deer population (82, 83). Small mammals provide additional mechanistic insight into amplification dynamics. La Crosse virus (LACV), the leading cause of pediatric arboviral encephalitis in the U.S., is maintained in enzootic cycles between *Aedes triseriatus* mosquitoes and small mammals, particularly chipmunks and squirrels, which develop sufficient viremia to support transmission (84). Climate-driven changes are now linked to shifts in their geographic ranges and seasonal activity, especially for tree squirrels, including gray and fox squirrels (85). These changes occur in response to warming and more arid conditions, leading to movement to higher elevations and altered hibernation and emergence timings (85). Such shifts modify spatial and temporal overlap with mosquito vectors, potentially altering contact rates within LACV transmission hotspots. Concurrently, climate-related stressors, such as heat, drought, and resource limitation, can affect population density and immune status, thereby modulating their capacity to sustain viremia and contribute to viral amplification (86, 87). The same is true for small insectivores like shrews and hedgehogs, whose high metabolic rates and dependence on moisture-rich habitats make them particularly responsive to temperature and environmental change. These species have been shown, through experimental and epidemiological studies, to act as amplifying hosts for SFTSV in endemic areas of China (66, 88, 89). Climate warming is now driving range shifts, altering hibernation patterns, and disrupting the seasonal activity of these mammals (88, 90).

Oropouche virus (OROV) in South America provides a complementary example of how amplifying host systems intersect with anthropogenic and climate-driven change to drive emergence. OROV, a midge-transmitted human pathogen, is maintained in enzootic cycles involving New World non-human primates (sloths, marmosets, and capuchins) and arthropod vectors (midges and mosquitoes), with early spillover events linked to deforestation and human encroachment into forested habitats. These processes increased contact between humans, amplifying hosts, and vectors, facilitating transmission into human populations. As climate change reshapes vector ecology and host distributions, and as land-use change and human mobility intensify, these interfaces are likely to expand. This expansion is expected to increase the frequency, geographic spread, and scale of OROV outbreaks (91, 92).

Together, these systems illustrate a unifying principle: climate change shapes host abundance, distribution, and physiology. In parallel, human-driven changes in land use, livestock movement, and ecosystem structure determine how these hosts interface with vectors and humans.

### Climate-sensitive vector dynamics

Besides hantaviruses, bunyaviruses depend on arthropods for their maintenance, transmission, and emergence (1, 93, 94). Though there are many examples of diverse arthropods contributing to the maintenance of bunyaviruses, such as mites (e.g., SFTSV [95, 96]; genus: *Bandavirus*), bed bugs (e.g., Kaeng Koi virus [95, 97]; genus: *Orthobunyavirus*), and bat flies (e.g., Wolkberg virus [95, 98]; genus: *Orthobunyavirus*), the primary vectors associated with the bunyavirus-human interface are ticks, mosquitoes, midges, thrips, and sandflies. These vectors impact agriculture, livestock, and human health and exhibit various climate-sensitive factors associated with their global distribution. Although this review does not focus on mechanisms, environmental conditions can directly influence vector competence and virus-vector interactions, acting alongside changes in vector behavior and geographic range to shape transmission risk. RVFV provides a well-studied example because of its clear environmental responsiveness (99). Mosquito susceptibility to RVFV infection is temperature-sensitive and species-specific, as *Cx. pipiens* shows reduced infection susceptibility at lower temperatures (100), whereas *Ae. fowleri* can transmit RVFV earlier after infection at higher rates when maintained at higher temperatures (101). Other studies have implicated vector-associated microbial communities as an additional temperature-sensitive factor shaping RVFV transmission dynamics. These microbial communities vary by host species, collection site, and short-term temperature change (102), with likely consequences for vector competence and virus transmission (103). For example, higher mean temperatures have been associated with lower abundance of *Wolbachia* in *Cx. pipiens* (104, 105). *Wolbachia* is a common endosymbiotic bacterium that can modulate arbovirus infection and transmission in mosquitoes, although its effects are context dependent and do not universally affect RVFV infection (106). Together, these examples highlight the complex interactions underpinning climate-sensitive vector-borne transmission. Although many of the factors may also affect insect-specific bunyaviruses, including members of the genera *Herbevirus* (107), *Orthophasmavirus* (108), and *Goukovirus* (109), we focus here on the bunyaviruses that interface directly with human and animal health.

#### Ticks

The role of ticks in the maintenance of bunyaviruses has been extensively studied due largely to the threat posed by priority pathogens within the *Bandavirus* and *Orthonairovirus* genera. A central ecological concern is the impact of climate change on habitat suitability, as warming temperatures and shifting seasonality reshape the geographic distribution of tick populations (4, 5). As regional climate-related impacts are inconsistent, the response of various tick species to this shift is not unidirectional. For example, various lifecycle stages of ticks are highly dependent on temperature and precipitation, and as these patterns shift on a regional level, so too does tick survival. Extensive climate modeling in relation to the shifting ranges of many clinically relevant tick species consistently reveals both contraction and expansion of predicted ranges in the coming decades, with the shared conclusion of a northern push in suitable climates (21, 79, 110–113). *H. marginatum*, one of the primary vectors of CCHFV, exemplifies this complexity. Broadly, this species is predicted to expand its range in the coming decades into climatically suitable regions across southern and central Europe, reflecting a general northward trend; however, this expansion is not expected to be uniform. Regions, such as central Turkey, are projected to experience decreased climatic suitability for *H. marginatum*, potentially due to increased drought conditions (112, 114). Similar patterns are observed among numerous other tick species inhabiting arid environments, including central and eastern Saudi Arabia (110), as well as regions with pronounced dry seasons across sub-Saharan Africa (115), including Zimbabwe (111) and South Africa (116). Climate models predict great regional heterogeneity and decreased habitat suitability for many species. Other species primarily distributed in temperate regions show more uniform predictions of future climate suitability. *Ixodes ricinus*, a widespread tick associated with several bunyaviruses (117), is predicted to expand into suitable regions across northern and eastern Europe (118). Similarly, *H. longicornis*, a vector of various bandaviruses across East Asia, Australia, and North America (119), is predicted to expand to suitable climates across southern and western Canada and shows increasing potential for establishment in parts of Europe (21). Additionally, as discussed above, migratory bird populations represent a potential long-range dispersal mechanism to these newly suitable climates (54, 56), as does the global livestock trade (120–122). These observations are intrinsically linked to tick activity and their development, as mild winters in northern climates lead to earlier active periods and increased survival, while increasing temperatures in southern regions lead to decreased humidity and thus decreased tick survival. These opposing effects are supported by historical and observational data (115, 123). Together, these dynamics highlight tick-borne bunyaviruses as particularly sensitive to environmental change, with shifting vector distributions and activity patterns creating new opportunities for transmission and geographic spread.

#### Mosquitoes

Due to the vast number of significant human pathogens spread by mosquito vectors worldwide (e.g., *Plasmodium* spp., Dengue virus, West Nile virus, Chikungunya virus, etc.) (124), the effects of climate change on their activity, range, and population dynamics are under regular study. As for bunyaviruses, various members of the *Orthobunyavirus* genus are important mosquito-borne viruses that affect both human (e.g., LACV [125, 126]) and livestock (e.g., CVV [127]) populations; however, RVFV, from the genus *Phlebovirus* (family *Phenuiviridae*)*,* is the best-characterized climate-sensitive mosquito-borne bunyavirus, causing recurrent outbreaks across sub-Saharan Africa and the Arabian Peninsula (128, 129). The unique features of its biology and lifecycle make the mosquito vector especially vulnerable to short-term ecological disruptions. Different mosquito species have different breeding behaviors that respond to water availability. In the classic, simplified view of RVFV epidemiology, the interplay between primary (*Aedes* spp.) and secondary (*Culex* spp.) mosquito vectors leads to rapid outbreaks following flooding. *Aedes* spp. vectors lay desiccation-resistant eggs in dry soil, which, upon heavy rainfall and flooding, hatch and multiply, while other vectors, including *Culex* spp. mosquitoes, lay eggs and multiply in the newly created floodwaters (99, 129–131). On the other climate extreme, drought conditions can force the interface of vectors and amplifying hosts around a limited number of water sources (132–134). These interactions can be exacerbated under conditions of “climate whiplash,” where repeated sudden shifts in weather conditions have synergistic effects that lead to emergence, spread, and outbreaks of mosquito-borne disease (135–137). These patterns are also implicated in RVFV outbreaks and their relationship to El Niño Southern Oscillation Events (138, 139). Aligning with the other vectors in this review, suitable climates for many mosquito species have expanded in recent decades and are predicted to expand further both latitudinally and altitudinally, bringing novel vectors into naïve regions, including those of RVFV and LACV (126, 140–142).

#### Midges

Another significant vector, especially for members of the *Orthobunyavirus* genus, is the *Culicoides* biting midge (Diptera: Ceratopogonidae) (95, 143). Though midge-borne bunyavirus infection of humans does occur, with the primary example being OROV and its reassortants (92, 144), these viruses more so impact ruminant populations across the globe, many of which are affected through teratogenic effects (145–148). The primary climate-associated factor impacting the biting midge lifecycle is a steady lengthening of the active season. Warming winter temperatures lead to earlier first emergence in spring, followed by a longer season of days above the developmentally suitable minimum temperature; for example, long-term seasonality surveillance (yearly first and last appearance) has revealed as much as a 40-day increase in the annual seasonal active period of *Culicoides* over 40 years of surveillance at some sites in the United Kingdom (149). This longer active period increases both the developmental rate and the number of generations before winter inactivity, thereby leading to an increased abundance of midges (149–153). Additionally, though their typical daily flight range is quite limited, their size facilitates long-range dispersal via wind currents and may allow the carriage of viruses into naïve regions (152, 154, 155). These wind patterns represent a potentially climate-sensitive ecological factor, with much uncertainty (4, 5, 156).

#### Thrips

Barring future ICTV taxonomic revisions, plant-infecting bunyaviruses are currently classified within three families: *Phenuiviridae*, *Tospoviridae*, and *Fimoviridae*. Among these, members of the genus *Orthotospovirus* (tripartite genome; family *Tospoviridae*) are of greatest agricultural importance. Unlike other plant-infecting bunyaviruses, orthotospoviruses are persistently transmitted by thrips, which serve as both vectors and amplifying hosts, feeding on and reproducing within a wide range of agricultural crops (157, 158). The prototype and namesake of this genus is tomato spotted wilt virus (TSWV), which has been ranked among the top 10 plant viruses of scientific and economic importance (second only to tobacco mosaic virus) (159). Since its first description in 1915 (160), TSWV has established a worldwide distribution driven largely by the ubiquity of its vectors and its exceptionally broad host range (over 1,000 plant species across over 90 plant families) (158, 161, 162). TSWV reemerged in the 1980s following the European introduction of its primary vector, Western flower thrips (*Frankliniella occidentalis*), and has since caused repeated severe outbreaks globally in many major crops, such as tomatoes, peppers, peanuts, potatoes, and lettuce, greatly reducing crop yield (up to 100%) and crop market value, with worldwide economic losses estimated to exceed $1 billion USD by 1994 (159, 161–164). Similar in impact but more limited in geographic range is groundnut bud necrosis virus, another orthotospovirus responsible for devastating agriculture in southern Asia (165). The relationship between TSWV and its vector is unique among plant viruses. In addition to being transmitted by thrips, TSWV replicates within both its plant host and vector species, making thrips more than simple carriers but instead integral, potentially co-evolved hosts (160, 166, 167). Given this dependence, climate-associated impacts on thrips are critical to global agricultural risk. As with other vectors on this list, the primary climate impacts on thrip populations are degree-day accumulation and overwintering potential. Degree-days represent the accumulation of heat above a developmental threshold, enabling insect growth, development, and reproduction (168). Unsurprisingly, as global average temperatures rise (4, 5) and regional climate patterns shift, degree-day accumulation is expected to increase. While rising temperatures generally accelerate thrip development, other factors, such as precipitation, exert more variable effects. For example, moderate and consistent precipitation can enhance plant growth and delay seasonal inactivity, whereas prolonged rainfall and flooding can reduce populations by killing the thrip larvae and suppressing the adult population (169–171). In addition, increasing global temperatures are associated with milder winters, which may enhance overwinter survival, increase carryover populations, and alter development cycles (172–175). Collectively, these trends suggest an overall increase in thrip activity and associated transmission risk, although the magnitude and direction of these effects will vary regionally.

#### Sandflies

Phlebotomine sandflies (Diptera: Psychodidae, Phlebotominae) are a diverse group of small, widely distributed, primarily nocturnal, blood-feeding insects. Their association with disease, in both humans and animals, is largely as the primary vector of the protozoan *Leishmania infantum*, the causative agent of leishmaniasis (175–177). Among arboviruses, the principal human pathogens transmitted by sandflies are the endemic bunyaviruses of the Mediterranean basin, including many members of the *Phlebovirus* genus, such as Toscana virus, sandfly fever Naples virus, and sandfly fever Sicilian virus (176, 178, 179). These febrile infections were well described among U.S. soldiers in Italy during World War II (180), although they were likely observed earlier (179, 181). Unlike tick and midge vectors, sandflies are limited by their lack of long-range dispersal mechanisms, such as avian transport, wind currents, or livestock movement. This constraint reflects their low stress tolerance, weak flight capacity, and the absence of clearly defined vertebrate amplifying hosts (182–187). However, despite these limitations, sandflies exhibit consistent, gradual shifts in their ecological range in response to climate change, particularly at northern latitudes. Climate modeling indicates that sandfly distribution is strongly constrained by winter minimum temperatures and moisture availability, both of which are steadily shifting in European regions north of the Mediterranean (183, 184, 188–190). Importantly, these patterns are region-specific. For example, in tropical regions, such as Sri Lanka, sandfly populations are predicted to decline under increased total sunshine exposure and higher ambient temperatures, underscoring the risk of overgeneralizing climate impacts at a global scale (191). In addition to geographic range, climate variability also influences sandfly activity patterns. Daily fluctuations in temperature, barometric pressure, and humidity can alter vector behavior, although these effects are species-specific (192–194). Notably, some species exhibit increased indoor activity during rainfall, suggesting both resilience to short-term environmental fluctuations and increased interface between humans and vectors (192, 195). Although sandfly climate sensitivity remains less studied than that of the other bunyavirus vectors, the overall trajectory is consistent: shifting environmental conditions are likely to expose previously unaffected human populations to sandfly-borne viruses.

### Climate-driven emergence patterns and ecological amplification

#### Habitat shifts and outbreak amplification

Climate-driven environmental changes reshape transmission dynamics by altering the spatial overlap and temporal synchrony of vectors and hosts, often producing episodic amplification events. These dynamics are frequently expressed as pulses of transmission following environmental perturbations. The 1993 SNV outbreak remains a canonical example of rainfall-driven amplification, in which El Niño-associated precipitation altered vegetation and rodent ecology, increasing spillover risk (37, 196). Similarly, RVFV outbreaks demonstrate how anomalous rainfall and flooding can synchronize vector emergence and host availability, producing large-scale epizootics and human outbreaks (131, 139), including major epidemics in Kenya (1997–1998) (197) and the widespread transmission of RVFV across the Horn of Africa (2006–2007) (198, 199). Consistent with these dynamics, heavy rainfall promotes mass hatching of infected *Aedes* eggs and the proliferation of secondary vector species, amplifying transmission potential (129). In parallel, habitat disruption and environmental variability can displace host and vector populations, increasing contact at the human-animal interface and further elevating spillover risk. In contrast, some bunyaviruses emerge through more gradual expansion rather than discrete amplification events. Heartland virus (HRTV) (200–202) exemplifies this pattern, with its distribution closely tracking the range of the Lone Star tick (*Amblyomma americanum*), whose expansion is supported by climatic suitability and increasing white-tailed deer populations that serve as key reproductive hosts (203). This alignment of vector persistence, host density, and human exposure highlights an alternative pathway to emergence driven by habitat-mediated range expansion.

#### Vector invasion and ecological restructuring

Invasive or expanding vector species can rapidly restructure transmission landscapes by altering host contact patterns and ecological niches. LACV transmission in the Appalachian region during the mid-1990s provides a clear precedent. Increased incidence coincided with the establishment and expansion of *Aedes albopictus* alongside the native *Aedes triseriatus*, driven by human introduction and environmental suitability. Forest fragmentation, peridomestic habitat formation, and the accumulation of standing-water containers further supported mosquito proliferation and increased contact with small-mammal reservoirs (125, 126, 204). Rather than the emergence of a novel virus, this reflects the ecological reconfiguration of an existing transmission system driven by vector expansion.

A similar concern arises with tick-borne bandaviruses. SFTSV is transmitted primarily by *Haemaphysalis longicornis*, a tick species that has recently expanded across multiple regions of the U.S. (205, 206). Its rapid geographic spread, high reproductive capacity, and broad host range illustrate how invasive vectors can create ecological conditions permissive for pathogen introduction (206). Although SFTSV has not been detected in the U.S., the presence of a competent vector raises the possibility that viral introduction through travel, animal movement, or migratory wildlife could enable local transmission. Climate-driven expansion of suitable tick habitat further increases the probability of such scenarios. Similarly, as described in “Avian hosts,” above, the introduction of *Hyalomma* spp. ticks into central Europe via migratory birds led to the establishment of this vector in the now-suitable climate and acts as a recent example of this process (56, 58). The invasion of these vectors has led to the establishment and subsequent expansion of CCHFV in countries such as Spain (54, 55, 207, 208).

#### Human amplification and urban emergence

Human-driven environmental change increasingly shapes where and how bunyaviruses emerge. The orthobunyavirus OROV has demonstrated rapid geographic expansion and increasing urban transmission across the Americas and the Caribbean. Expansion into densely populated settings reflects the convergence of vector redistribution, human movement, and land-use change. The Amazon basin provides climatic conditions highly favorable for vector persistence, and epidemiological analyses link OROV incidence to temperature, precipitation, and vegetation indices (209), within a region recognized as climatically vulnerable (210, 211). Deforestation and urbanization increase human-vector contact, while socioeconomic vulnerability influences exposure risk and outbreak severity (212). As geographic range expands, the likelihood of co-circulation of distinct viral lineages within shared vector and host populations also increases. In this context, OROV illustrates how climate-associated environmental change, combined with urbanization and human movement, can influence both transmission dynamics and evolutionary opportunity. Emerging genomic data also suggest constant reassortment and potential adaptation among circulating lineages (213), highlighting how ecological convergence may intersect with the segmented genome architecture of bunyaviruses to generate novel viral constellations. More broadly, urbanization also drives vector adaptation, as species, such as *Ae. aegypti*, exploit urban breeding habitats and stable infrastructure, increasing the human-vector interface contact while shifting vector behavior and feeding preferences (214–218).

## AUTHORS’ PERSPECTIVE

Climate change is a powerful modifier of infectious disease dynamics, particularly for vector-borne viruses whose complex transmission cycles depend on the finely balanced interactions among vector, vertebrate host, and pathogen. However, the effects of climate change are neither uniform nor linear (219). While warming temperatures and altered precipitation patterns can expand vector ranges and prolong transmission seasons, ecological constraints may simultaneously limit expansion in other regions. For example, modeling studies predict that decreasing the abundance of total bird populations in Europe may reduce their projected contribution to tick range expansion (220). Similarly, disease burden associated with *Aedes albopictus* is expected to decrease in parts of the tropics where temperatures exceed the mosquito’s optimal thermal range, pushing conditions outside its ecological suitability window (140). These findings highlight that climate suitability operates within defined ecological bounds, and conditions outside these ranges may reduce rather than increase transmission. OROV illustrates this uncertainty. Despite decades of circulation in parts of South America, OROV reemerged explosively between late 2022 and 2025 across new geographic areas, including regions previously considered outside sustained transmission zones (92). The drivers of this resurgence remain incompletely defined and likely reflect a convergence of human migration, land-use change, climate variability, and viral evolution (92). Although OROV has not been isolated from birds, antibodies detected in more than 11 avian families in Brazil raise the possibility that shifts in avian host competence and/or vector ecology could reshape its transmission dynamics (92, 144, 221). The role of mosquitoes in OROV transmission also remains unresolved. Several species, including *Cx. quinquefasciatus*, *Cx. tarsalis*, and *Ae. albopictus,* have shown limited vector competence for OROV (222, 223), and some *Culex* species feed primarily on avian hosts (224). This overlap suggests a potential, but still unconfirmed, route by which OROV could interface with avian populations. Whether OROV follows a trajectory of continued geographic expansion, as seen with West Nile virus (225, 226), or stabilizes within ecological constraints, remains uncertain. What is clear, however, is that bunyaviruses are highly sensitive to climate and anthropogenic environmental change. The complexity of their transmission systems limits precise forecasting, but this same complexity defines where predictive frameworks must improve. Anticipating future risk will require approaches that integrate climate, ecological, and evolutionary data rather than relying on single-factor projections. Preparedness strategies must therefore be built around uncertainty, focusing on adaptable surveillance and response systems rather than assuming uniform expansion.

## STRATEGIC SURVEILLANCE AND PATHOGEN PRIORITIZATION IN A CHANGING CLIMATE

If bunyavirus emergence is shaped by complex, climate-sensitive ecological systems, then surveillance strategies must be designed to detect and interpret these dynamics before large-scale outbreaks occur. Despite their public and veterinary health importance, bunyaviruses remain underrepresented in global surveillance efforts compared with other arboviruses. Diagnostic limitations, sparse genomic sequencing, and inconsistent reporting hinder early detection of emergence events. In many regions, bunyavirus infections are misdiagnosed or go unrecognized, particularly when their clinical presentations overlap with those of more familiar febrile illnesses, such as dengue or chikungunya (92). These gaps are likely to be amplified under ongoing climate and anthropogenic change, particularly as vectors expand into regions with limited diagnostic capacity, minimal historical awareness, and immunologically naïve populations (1, 6, 92, 140, 219, 227, 228). Without surveillance systems that integrate climate projections, vector monitoring, seroepidemiology, and real-time viral genomics, predictive modeling will remain fragmented and reactive (229, 230).

OROV’s expansion across the Americas illustrates how long-circulating pathogens can rapidly shift geographic range when ecological and climatic conditions change (92). In this context, the coordinated roadmap developed by the World Health Organization (WHO) and the UK Health Security Agency (UKHSA) (231) provides an important foundation for bunyavirus preparedness. However, translating strategic guidance into operational impact will require sustained investment in longitudinal surveillance, expanded genomic sequencing capacity, standardized case definitions, and cross-border data integration. A forward-looking preparedness strategy should therefore prioritize:

1. systematic climate-informed vector surveillance,
2. scalable diagnostics that distinguish bunyaviruses from other co-circulating arboviruses,
3. routine genomic monitoring to detect reassortment and adaptive changes, and
4. integrated One Health frameworks that unify human, animal, and environmental data sets.

An integrated One Health framework is essential to this effort (232). Most bunyaviruses emerge from animal reservoirs and are shaped by ecological forces that regulate vector abundance and human exposure. Surveillance restricted to human case detection captures only the final link in the transmission chain. Linking human clinical data with animal surveillance and environmental monitoring, including vector dynamics, land-use change, and climate variability, enables the detection of shifts in transmission intensity before sustained human amplification occurs. Alignment across veterinary, ecological, and public health systems, therefore, supports proactive, risk-targeted responses rather than reactive outbreak management.

International policy frameworks increasingly recognize that cross-sectoral surveillance is foundational to early detection and response in arboviral systems (233). A pragmatic path forward is to prioritize high-consequence bunyavirus pathogens (234–236) and develop predictive models centered on climate-sensitive shifts in vector distribution, viral evolution, and human contact patterns. While reservoir hosts remain incompletely defined for several bunyaviruses, this uncertainty should not paralyze preparedness efforts. Focusing on the vector-virus-human interface provides a tractable and biologically coherent framework for forecasting emergence across diverse ecological contexts. By projecting which viruses are more likely to expand geographically, undergo adaptive change, or increase spillover frequency, surveillance can move from descriptive monitoring to anticipatory risk assessment.

## CONCLUSIONS

While the climate-virus discourse has largely centered on the flavi- and alpha-viruses, bunyaviruses represent a significant group of pathogens whose emergence risk may be especially responsive to ecological disruption. Their reliance on environmentally sensitive transmission networks, combined with the evolutionary flexibility conferred by genome segmentation, positions them at the intersection of environmental change and viral adaptation. Nearly two decades after early calls to consider the implications of climate change for bunyaviruses, the evidence base has expanded, but so too has the scale and pace of anthropogenic environmental change. The challenge is no longer theoretical. Anticipating and mitigating climate-sensitive bunyavirus emergence will require sustained investment in genomic surveillance, integrated vector monitoring, and experimental systems capable of interrogating virus-host-vector interactions under shifting environmental conditions. Climate does not act in isolation but in concert with land use change, urbanization, and shifting host and vector distributions to amplify existing ecological and evolutionary processes. Recognizing and responding to this amplification will be central to reducing the future burden of bunyavirus-associated disease.

## EDITOR'S NOTE

**Ed. Note:** A potential conflict of interest was identified after acceptance of this paper, and the editor in chief conducted an additional review to ensure the integrity and fairness of the peer review process.
